# Effects of high-pressure homogenization on physicochemical and functional properties of enzymatic hydrolyzed soybean protein concentrate

**DOI:** 10.3389/fnut.2022.1054326

**Published:** 2022-11-24

**Authors:** Yaru Liang, Yanan Guo, Yuxuan Zheng, Sibo Liu, Tianfu Cheng, Linyi Zhou, Zengwang Guo

**Affiliations:** ^1^College of Food Science, Beijing Technology and Business University, Beijing, China; ^2^College of Food, Northeast Agricultural University, Harbin, Heilongjiang, China; ^3^Key Laboratory of Soybean Biology, Ministry of Education, Northeast Agricultural University, Harbin, Yunnan, China

**Keywords:** high-pressure homogenization, Alcalase protease, soybean protein concentrate, physicochemical properties, functional properties

## Abstract

This paper investigates the effect on the physicochemical and functional properties of soybean protein concentrate (SPC) by using Alcalase protease and high-pressure homogenization (HPH) (0, 20, 40, 60, 80, and 100 MPa) for the combined modification. The results showed that the degree of hydrolysis of SPC was 4.1% and the antigen protein was degraded after Alcalase hydrolysis, when the homogenization pressure (HP) was 6 0Mpa, the particle size of the SPC was the smallest, the zate potential absolute value up to 33.45 mV, the secondary structure has the lowest β-sheet content, the highest random coil content, and the highest surface hydrophobicity (H_0_), the size of protein fragments on the microstructure surface is the smallest, the lowest denaturation temperature (T_*d*_) and enthalpy (△H) are 72.59°C and 1.35 J/g, the highest solubility is 80.54%, and the highest water and oil holding capacities are 7.73 g/g and 6.51 g/g, respectively. The best emulsifying activity and emulsifying stability were 43.46 m^2^/g and 190.35 min, the most even distribution of emulsion droplets. This indicates that the HPH treatment destroys the structure of enzymatic hydrolyzed SPC, changes its physicochemical properties, and improves its functional properties. In this study, SPC was modified by HPH and enzyme combined treatment, in order to improve the functionality and application range of SPC, and provide a theoretical basis for its high-value utilization in the food field.

## Introduction

China is rich in soybean resources, which is considered as a high-quality source of plant protein. Protein, the main component, accounts for about 40% of soybeans. Soybean protein is rich in amino acids required by humans, making soybean protein the best vegetable protein to replace animal protein and is usually consumed directly or indirectly as a food additive in production.

Soybean protein has many excellent functional properties and has different applications in different food processing. The amino acid compositions of 7S and 11S are different, and the protein spatial structures formed are different. Therefore, 7S and 11S have different characteristics in functional properties (1). Because of its low price, high nutritional value and complete functions, soybean protein can be used as a good source of functional food. Therefore, a good study of the functional properties of soybean protein can better use its high cost performance ratio to benefit mankind (2). The quality and sensory physicochemical properties of soybean protein are called functional properties of soybean protein (3). Soybean protein concentrate (SPC) has a protein content of about 70%, which is lower in price than soybean protein isolate. It contains a lot of essential fatty acids, phospholipids and minerals such as calcium and phosphorus that are beneficial to the human body, as well as 9 essential amino acids. Its amino acid composition is close to animal protein, and the digestion utilization rate is as high as 90%, which has high nutritional value (4). Among legumes, soybean is the most commonly used protein source because of its high protein content. Soybean products contain large amounts of crude protein and are rich in essential amino acids for humans: 70% of this is in concentrate form and 90% in isolate form. Therefore, the production of soybean protein hydrolysate is very promising. Bioactive peptides derived from soybean protein have dual roles in improving health-related functions and improving the technical properties of foods (5).

Soybean protein concentrate (SPC) obtained from the extraction of carbohydrate and lipid from soybean meal is a promising source of vegetable protein, which can be used to replace animal protein in aquatic feed and produce meat analogs (6, 7). And soybean protein concentrate has the advantages of non-polluting emissions, lighter flavor, good appearance, low isoflavones, oligosaccharides, etc., and has a lower price than soybean protein isolate, but its functional properties are poor quantity is limited (8, 9).

The antigenic protein contained in soybean protein leads to a decrease in its feed utilization rate. As one of the soybean protein products, SPC also contains antigenic proteins. The extensive use of SPC in animal feed can cause digestive stress reactions such as intestinal allergy and diarrhea, resulting in lower feed digestibility (10), which limits its application in the field of food processing to a certain extent. The results show that bioenzymatic hydrolysis can be performed selectively by different enzyme preparations, which is considered to be an effective method to reduce or even completely eliminate the antigenicity of soybean protein (11). Studies have shown that the hydrolysis of SPC by Alcalase protease can not only eliminate the antigenicity of soybean protein but also reduce the production cost (12). However, due to the low degree of hydrolysis of the SPC product after enzymatic hydrolysis, the surface hydrophobicity, emulsification, gelation and other properties of SPC are not significantly improved. It is found that high pressure homogenization can improve the functionality of food protein by forming soluble aggregates (13). Wang et al. (14) showed that the heat-induced gelation performance of the protein decreased after being subjected to high-pressure homogenization at 200-600 Mpa. Molina (15) and other studies found that soybean protein 7S can achieve the highest emulsification and surface hydrophobicity after 400 Mpa pressure treatment, while 11S can achieve the highest emulsification and surface hydrophobicity after 200 Mpa pressure treatment.

Therefore, on the basis of previous research by experts and scholars, in order to improve the functionality and application range of SPC, so that it can be used at a high value, this study uses Alcalase protease to hydrolyze SPC, and then uses HPH to modify the SPC after enzymatic hydrolysis. It provides a theoretical basis for its high value utilization in the food field.

## Materials and methods

### Materials and reagents

Soybean protein concentrate (SPC) Kedong Yuwang Soybean Protein Food Co., Ltd., Alcalase protease (Vitality 1.0 × 10^5^U/g) Novozymes, China, SDS-PAGE gel electrophoresis kit Harbin Clover Biotechnology Co., Ltd., Dithiothreitol (DTT), Sodium tetraborate, potassium bromide, bovine serum albumin, ethanol American sigma company, Arowana soybean oil purchased from local supermarkets.

### Sample preparation

#### Alcalase protease hydrolysis

Referring to the research method of Cui et al. (16), the SPC was enzymatically hydrolyzed antigen protein. The parameters are set as follows: the ratio of material to water is 1:1.5, the addition amount of Alcalase protease is 0.8%, pH = 6.5, time 2 h, and the temperature is 50°C. After the reaction, the enzyme was inactivated in a boiling water bath, the samples were dried at 80°C, and the degree of hydrolysis and degradation effect of SPC were measured after pulverization.

#### Determination of proteolysis degree and degradation effect of antigen

The degree of hydrolysis of SPC was determined with reference to the P.M. Nielsen (17) ortho-phthalaldehyde (OPA) method. OPA reagent (prepared and used now): First completely dissolve 7.620 g sodium tetraborate and 200 mg SDS into 150 ml distilled water, then dissolve 160 mg 97% OPA into 4 ml ethanol, mix and add 176 mg 99% DTT to the solution, and set the volume to 200 ml with distilled water.

The sodium dodecyl sulfate-polyacrylamide gel electrophoresis (SDS-PAGE) test was carried out with reference to the method of Laemmli (18). The 12% separation gel and 5% concentration gel are combined to form the acrylamide gel required for the experiment. Take 50 μl 10 mg/ml SPC solution and 10 μl fully mix the loading buffer solution. Boil the mixture in boiling water for 5 min, centrifuge the sample at 8,000 r/min for 20 min. Take 20 μl sample add to each swimming lane, set the running value of electrophoresis instrument protein swimming lane, concentrate gel 80 mV, separate gel 120 mV, put the gel into the dye solution in the kit after the operation stops, and dye for 30 min. Then transfer the gel to the decolorizing solution to decolorize for 12 h until the protein swimming lane appears. After gel imaging, the electrophoresis images were analyzed with ImageLab (Bole, USA) software.

#### High-pressure homogenization treatment of hydrolyzate

The hydrolyzate was processed at 0, 20, 40, 60, 80, and 100 Mpa using an experimental high-pressure homogenizer (AXA United Technologies, UK) for three cycles. The prepared samples were put into a Christ freeze dryer (Beijing Aochuang Xingye Co., Ltd.) to obtain a modified soybean protein concentrate powder, which was sealed in a desiccator for later use.

### Determination of physicochemical properties of soybean protein concentrate (SPC)

#### Determination of particle size

With reference to the methods of Zhang et al. (19), it is slightly modified. Sizer Nano ZS90 particle size analyzer was used to determine the particle size of SPC. The sample solution was prepared with a concentration of 1 mg/10 ml, an observation Angle of 173°, a refractive index of 1.333 and a viscosity of 0.00088 Pa.s. The value of pure water at room temperature is the control.

#### Determination of zate potential

With reference to the methods of Liu et al. (20) it is slightly modified. The clear and transparent sample solution was prepared and the zeta potential was measured at 25°C using a Nano ZS90 particle size and potential analyzer (Malvern Malvern Instruments Ltd., UK).

#### Determination of secondary structure

With reference to the methods of Zhou et al. (21), slightly modified. Secondary structure characteristics of SPC were determined by Scimitar 2,000 Fourier transform infrared spectroscopy (Agilent Corporation, America). The lyophilized samples were thoroughly mixed with dried potassium bromide at 1:100 (m/m) and pressed into 1-2 mm sections. Spectra were acquired in 64 scans with a resolution of 8 cm^–1^ in the range of 400-4,000 cm^–1^. Fitting analysis of infrared spectral data was performed using Peakfit Version 4.12 software.

#### Determination of surface hydrophobicity

With reference to the method of Kato (22), the H_0_ of SPC was measured by ANS fluorescent probe method. SPC samples were dissolved in 0.01 mol/L, pH = 7.0 buffer to make 0.005, 0.01, 0.02, 0.1, 0.2% (w/v) solutions. Take 4 ml of sample solution, add 50 μl of 8 mmol/L ANS, shake well, and let stand for 3 min at room temperature. FL8500 Fluorescence (PE Instruments Inc., USA) emission spectrum test conditions: 290 nm is the excitation light wavelength, the scanning range is 300-400 nm, the excitation slit width and the emission slit is 5 nm to measure the fluorescence intensity. Taking the protein mass concentration as the abscissa and the fluorescence intensity as the ordinate, a curve was made, and the slope at the initial stage of the curve was the H_0_ of the protein.

#### Determination of scanning electron microscopy

With reference to the method of Zhang et al. (19), slightly modified. Field emission scanning electron microscope (Hitachi, Japan) was used to observe the surface microstructure of SPC. The sample is glued to the objective table and coated with a conductive layer, Parameter setting: 20 kV accelerating voltage, 4000X amplification.

#### Determination of thermal stability

With reference to the method of Yi et al. (23), slightly modified. Differential scanning calorimetry was measured the thermal stability of SPC. The 6 mg sample was sealed in an aluminum disk and heated from 20°C to 150°C at 10°C/min under N_2_ (40 mL/min), and the empty aluminum disk was used as a control.

### Determination of functional properties of soybean protein concentrate

#### Determination of solubility

With reference to the method of Yu et al. (24), slightly modified. Prepare a 2% (w/w) protein solution and place it in a centrifuge tube, THERMO X1R refrigerated high-speed centrifuge (Thermo Company, USA) processing conditions: 10,000 rpm, 30 min, 25°C, and take the supernatant. The crude protein content of supernatant was measured (25), and the curve was drawn with bovine serum protein as the standard sample. Sample solubility formula:


$$\mathrm{Solubility}\left(\%\right)=\frac{\mathrm{Protein}\,\mathrm{content}\,\mathrm{in}\,\mathrm{supern}\,\text{atant}}{\mathrm{Total}\,\mathrm{Protein}\,\mathrm{Content}}\times 100\%$$

#### Determination of water and oil holding capacity

According to the methods of Valdez-Hurtado et al. (26), it was slightly modified. The 2.5 g (M_1_) sample was diluted to 25 mL in deionized water, placed in a centrifuge tube, and centrifuged at 4°C, 8000 rpm, for 15 min. After centrifugation, the weight of the residue was recorded as M_2_ (g). Water holding capacity (WHC) formula:


$$\text{WHC}\,\left(g/g\right)=\frac{{\text{M}}_{2}-{\text{M}}_{1}}{{\text{M}}_{1}}$$

Where, M_1_ and M_2_ are the weight (g) of the sample before and after water absorption, respectively.

A total of 1.5 g (m_1_) of sample was mixed with 7.5 mL soybean oil and placed in a centrifuge tube for 12 h. Centrifugation was performed at 8,000 rpm for 20 min. The supernatant was discarded and the weight of residual was m_2_ (g). Oil holding capacity (OHC) formula:


$$\text{OHC}\,\left(g/g\right)=\frac{{\text{m}}_{2}-{\text{m}}_{1}}{{\text{m}}_{1}}$$

Where, m_1_ and m_2_ are the weight (g) of the sample before and after oil absorption, respectively.

#### Determination of emulsification activity and emulsification stability

With reference to the method of Pearce et al. (27), slightly modified. An emulsion was obtained by mixing 15 ml of the 0.1% (w/v) sample solution with 5 ml of sunflower oil by coarse homogenization at 10,000 rpm for 3 min. Take 50 μL of the emulsion at time intervals of 0 and 30 min from the bottom of the vessel and dilute it 100-fold with 5 ml of 0.1% SDS (sodium dodecyl sulfate). Measure the absorbance of the diluted solution with a UV-2700 spectrophotometer (Shimadzu Company, Japan) at a wavelength of 500 nm. All assays were performed in triplicate. Emulsifying Activity Index (EAI) and Emulsifying Stability Index (ESI) formulas:


$$\mathrm{EAI}\,\left({\text{m}}^{2}/g\right)=\frac{2\times 2.303\times {\text{A}}_{0}}{0.25\times \text{m}}$$

$$\text{ESI}\,\left(\text{min}\right)=\frac{{\text{A}}_{0}}{{\text{A}}_{0}-{\text{A}}_{30}}\times 30\,m\,i\,n$$

In the formula, A_0_ and A_30_ are the absorbance of the diluted sample emulsion at 0 and 30 min, respectively, and m is the weight of the sample (g).

#### Determination of confocal laser scanning microscope

The method of Zhang (19) was slightly modified, and the microstructure of SPC emulsion was observed by A1Si confocal laser scanning microscope (CLSM) (Nikon Corporation, Japan). Prepare 1% Nile blue and 0.1% Nile red dissolved in isopropanol, respectively, and then filter the Nile red and Nile blue to remove the residue. Use a pipette to pipette 200 μl of the emulsion prepared in 1.5.3, dilute it 5 times, add 55 μl Nile blue and 50 μl Nile red, mix well, make tablets after 0.5 h, and protect from light throughout the process. After the sample was stained, it was placed on a watch glass, placed in a laser confocal microscope, and photographed with a 10X eyepiece and a 60X objective lens at 552 and 488 nm wavelengths.

### Statistical analysis of data

All samples were tested in triplicate. SPSS 20.0 was used for statistical analysis, Origin 9.0 was used for data mapping, Peakfit Version 4.12 was used for fitting analysis. The mean value is expressed as ± SD. *P* < 0.05 indicated significant differences.

## Results and analysis

### Analysis of hydrolysis degree and sodium dodecyl sulfate-polyacrylamide gel electrophoresis of soybean protein concentrate hydrolyzed by Alcalase protease

**FIGURE 1:**
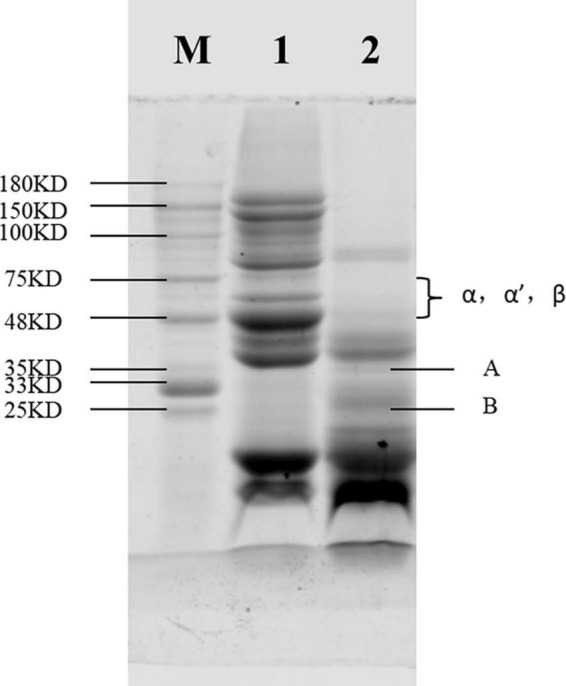
Sodium dodecyl sulfate-polyacrylamide gel electrophoresis (SDS-PAGE) of soybean protein concentrate (SPC) after Alcalase protease hydrolysis. Note: In the figure, M is the standard protein, 1 express the unhydrolyzed SPC, and 2 express the hydrolyzed protein.

Degree of Hydrolysis (DH) refers to the percentage of peptide bonds h (mmol⋅g^–1^ protein) broken in the total peptide bonds htot (mmoL⋅g^–1^ protein) of a given protein during protein Hydrolysis. The degree of hydrolysis of SPC was 13.5% after Alcalase hydrolysis by OPA. It can be seen from Figure 1 that due to the low degree of hydrolysis, SPC still has large molecular weight bands after hydrolysis, indicating that protein peptide is not only obtained after hydrolysis of soybean protein concentrate, but also protein structure exists.

Sodium dodecyl sulfonate polyacrylamide gel electrophoresis (SDS-PAGE) is a common method for quantifying, comparing and identifying proteins, which is economical, rapid and repeatable. The subunit structure of protein can be observed by SDS-PAGE. As shown in Figure 1, the α, α′ and β bands of the 7S protein of SPC were completely degraded after enzymatic digestion compared with the SPC without protease hydrolysis, and the basic polypeptide A and acidic polypeptide B bands of 11S were also not obvious, indicating that the antigenic protein in SPC was hydrolyzed. This may be because Alcalase protease hydrolyzes protein macromolecular bonds and exists in the state of small molecular free radicals, so the bands of large molecular weight become less obvious or even disappear, and the bands of small molecular weight increase.

### Effect of high-pressure homogenization on physicochemical properties of enzymatic hydrolyzed soybean protein concentrate (SPC)

#### Analysis of particle size

**FIGURE 2:**
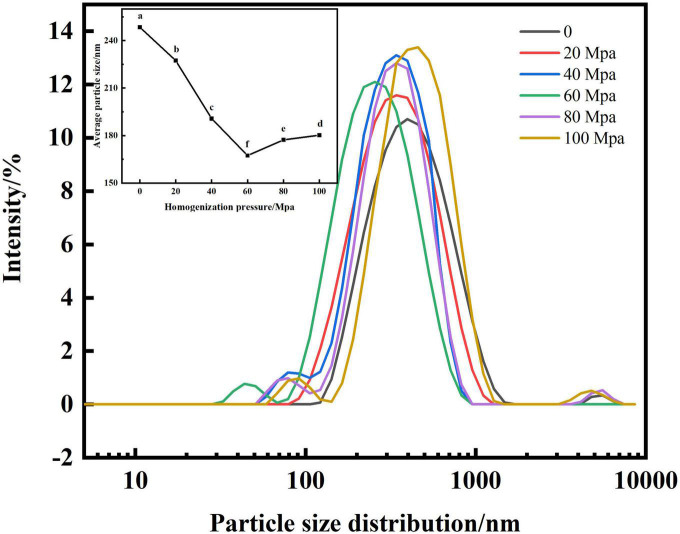
Effect of high-pressure homogenization (HPH) on particle size of enzymatic hydrolyzed soybean protein concentrate (SPC). Note: Different letters in the figures indicate significant differences (*P* < 0.05), the same below.

Figure 2 shows that the HPH treatment shifted the main peak of the particle size distribution of SPC to the left and significantly reduced the mean particle size compared with the untreated sample after enzymatic digestion. When the HPH treatment pressure [hereinafter referred to as: homogenization pressure (HP)] is 60 Mpa, the main peak of SPC appears between 100 and 1,000 nm, the impurity peak appears between 10 and 100 nm, and the average particle size is the smallest. When the HP is greater than 60 Mpa, the main peak of the particle size distribution of SPC shifts to the right, and there are stray peaks between 100 and 1,000 nm, and the average particle size also increases significantly. This may be because the HPH process can provide the energy required for emulsification to break the droplets into nano-droplets by generating strong turbulence, vibration, cavitation and hydraulic shear (28), thereby reducing the particle size of SPC. When the homogenization pressure is greater than 60 Mpa, the hydrophobic interaction between proteins may be weakened and aggregated due to excessive pressure (21), thus increasing the particle size of SPC. The results indicated that the structure of SPC was destroyed into nano-droplets after enzymatic hydrolysis by HPH, which changed the particle size of protein molecules.

#### Analysis of zate potential

**FIGURE 3:**
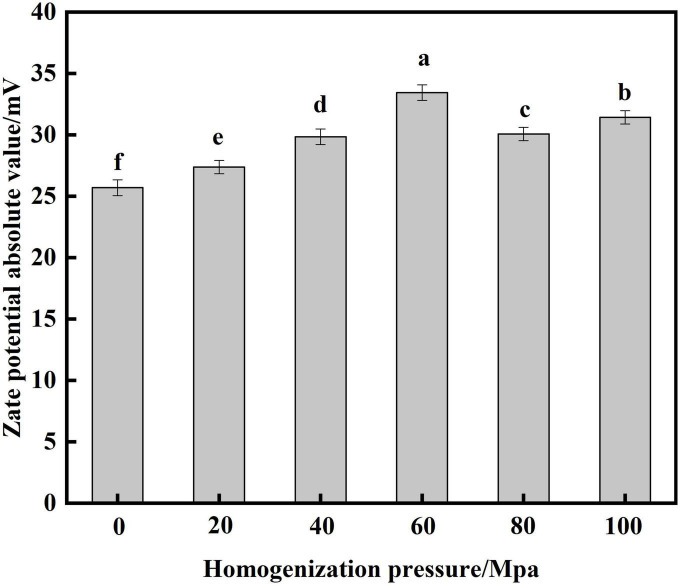
Effects of high-pressure homogenization (HPH) on the zate potential of enzymatic hydrolysis soybean protein concentrate (SPC).

Zate potential is an indicator that reflects the surface charge density of proteins and evaluates the stability of emulsion systems (29). Figure 3 show that the absolute value of zate potential of SPC increased significantly after HPH treatment compared to the untreated sample after enzymatic digestion. With the increase of HP, the potential showed a trend of first increasing and then decreasing. When the HP is 60 Mpa, the absolute value of the zate potential is at most 33.45 mV. This may be because the HPH treatment provides a larger energy barrier between the protein droplets, which provides a good electrostatic repulsion, thereby increasing the absolute value of the potential (21). When the HP is too large, the absolute value of the potential decreases, and studies have shown that electrostatic repulsion plays an important role in preventing protein aggregation (30). This may be because the aggregation of proteins, which reduces the electrostatic repulsion between proteins, resulting in a decrease in the absolute value of the potential of SPC. The results showed that the electrostatic repulsion and absolute zate potential of SPC changed after HPH, which affected the stability of the solution.

#### Analysis of secondary structure

Infrared spectroscopy can be used to study the molecular structure of proteins and their stability (31). From Table 1 that the untreated samples after enzymatic hydrolysis have the highest β-sheet content and the lowest random coil content. With the increase of HP, the content of α-helix and β-sheet tended to decrease and then increase, and the β-turn and irregular curl tended to increase and then decrease. When the HP was 60 Mpa, the β-sheet content was the lowest at 23.26% and the random coil content was the highest at 20.17%, indicating that the structure of SPC was scattered and disordered and the stability was low under this HP treatment. This may be because the fact that the cavitation and shear force and disruption of the HPH break the intermolecular bonds (32), thereby increasing the random coils in the secondary structure, increasing the disordered structure, and reducing the content of β-sheets. When the HP was too high, the content of β-sheets increased, probably due to the cross-linking reaction between proteins and the increase of intermolecular forces. It is shown that protein secondary structure is positively correlated with the interactions between different parts of the molecule (33). These results indicated that the HPH destroyed the intermolecular bonds in the secondary structure of SPC after enzymatic hydrolysis, which changed the intermolecular force and resulted in the change of the secondary structure of protein.

**TABLE 1:** Effects of high-pressure homogenization (HPH) on the secondary structure of enzymatic hydrolyzed soybean protein concentrate (SPC).

HPH (Mpa)	α-helix (%)	β-sheet (%)	β-turn (%)	Random coil (%)
0	20.01 ± 0.09^b^	31.24 ± 0.25^a^	33.48 ± 0.07^d^	15.27 ± 0.08^e^
20	17.32 ± 0.08^d^	30.72 ± 0.20^b^	32.29 ± 0.05^e^	19.67 ± 0.07^c^
40	14.41 ± 0.10^f^	26.32 ± 0.20^c^	40.05 ± 0.09^b^	19.22 ± 0.08^d^
60	15.72 ± 0.08^e^	23.26 ± 0.16^e^	40.85 ± 0.08^a^	20.17 ± 0.06^a^
80	20.70 ± 0.10^a^	25.60 ± 0.22^d^	33.66 ± 0.04^d^	20.04 ± 0.07^b^
100	19.55 ± 0.10^c^	26.36 ± 0.19^c^	34.15 ± 0.04^c^	19.94 ± 0.05^b^

Different letters in the data in the same column indicate significant differences (*p* < 0.05), the same below.

#### Analysis of surface hydrophobicity

**FIGURE 4:**
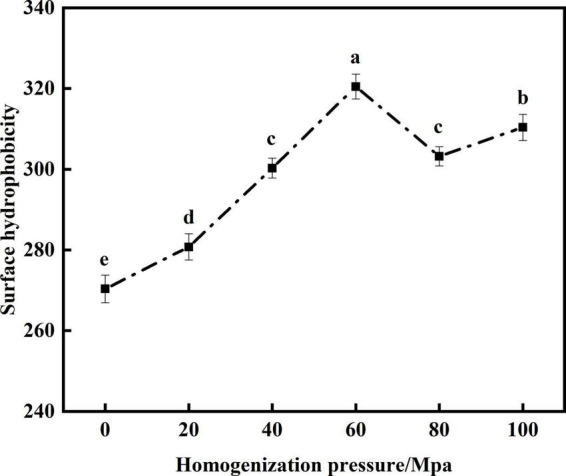
Effects of high-pressure homogenization (HPH) on the H_0_ of enzymatic hydrolyzed soybean protein concentrate (SPC).

The hydrophobic interaction between protein molecules is the main force that maintains its structure. H_0_ is the number of non-polar groups on the surface of a protein that come into contact with polar solutions, thereby affecting its emulsifying properties (34). From Figure 4 that compared with the untreated samples after enzymatic hydrolysis, the H_0_ of SPC was significantly increased after HPH. With the increase of HP the surface hydrophobicity increased first and then decreased. When the HP is 60 Mpa, the surface hydrophobicity of SPC is the highest. This may be because the HPH shear force exposed the SPC hydrophobic groups originally hidden in the folded structure, making the structure of the protein more flexible and H_0_ increased (35). However, when the HP is too high, the decrease of surface hydrophobicity may be because the increased shear force will lead to the aggregation of proteins through hydrophobic interaction, and the protein aggregates cover the hydrophobic water points on the protein surface, which limits the binding with ANS and reduces the surface hydrophobicity of proteins (36). The results showed that the HPH opened the protein structure after enzymatic hydrolysis and changed the exposure degree of hydrophobic groups, thus leading to the change of H_0_.

#### Analysis of scanning electron microscope

**FIGURE 5:**
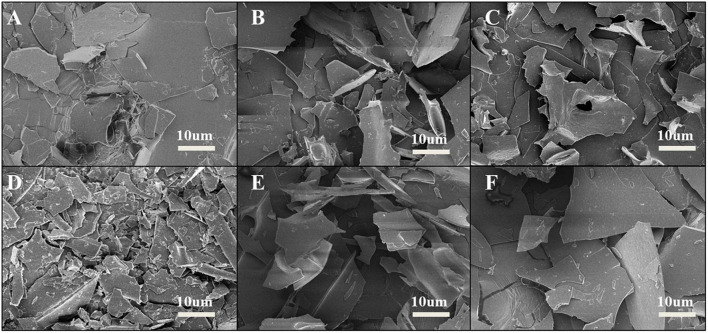
Effects of high-pressure homogenization (HPH) on the microstructure of enzymatic hydrolyzed soybean protein concentrate (SPC). Note: Letters **(A–F)** express 0, 20, 40, 60, 80, 100 Mpa of HPH after enzymatic hydrolysis.

Scanning electron microscope (SEM) was used to observe the surface microstructure of SPC treated by HPH after enzymatic hydrolysis. From Figure 5 that compared with the untreated sample after enzymatic hydrolysis, more fine protein fragments appeared on the surface of SPC after HPH treatment, and pores appeared on the surface. When the HP was 60 MPa, the protein fragment size was the smallest and the most abundant at the same magnification. This may be because the strong pressure generated by HPH combined with cavitation, inertia and shear forces, which broke the intermolecular bonds of proteins, leading to the fragmentation of large droplets into small droplets, the smaller particle size of proteins, and the formation of more small fragments after freeze-dried (37, 38). When the HP is too high, the protein surface debris particles are reduced and the surface is flat. This may be because the HPH treatment reduces the electrostatic repulsion between proteins and causes the cross-linking reaction to form large droplets (39), which leads to the reduction of protein debris particles and the formation of relatively flat surface. The results indicate that the HPH caused changes in the droplet size of the protein solution after enzymatic digestion, which led to changes in the microstructure of the freeze-dried protein surface.

#### Analysis of thermal stability

Research shows that proteins with higher T_*d*_ and ΔH reflect better thermal stability (40). From Table 2 that compared with the untreated samples after enzymatic hydrolysis, the T_*d*_ and ΔH of SPC by HPH treatment decreased first and then increased. When the HP was 60 Mpa, the T_*d*_ and ΔH of SPC were the lowest. This may be due to the fact that the protein structure is opened after enzymatic hydrolysis, and the internal structure of the protein is destroyed by HPH (41). The T_*d*_ and ΔH of the protein are related to its spatial structure (42), resulting in a decrease in T_*d*_ and ΔH during heating. When the HP is too high, more energy is required to denature the protein molecules due to the increased β-sheets content in the protein secondary structure (43), resulting in higher T_*d*_ and ΔH. The results indicate that the HPH causes changes in the spatial structure of the enzymatically digested proteins, leading to changes in their stability during the heating process.

**TABLE 2:** Effects of high-pressure homogenization (HPH) on the denaturation temperature (T_*d*_) and enthalpy (△H) of enzymatic hydrolyzed soybean protein concentrate (SPC).

HPH (Mpa)	T_*d*_ (°C)	△H(J/g)
0	81.47 ± 0.09^a^	3.41 ± 0.02^a^
20	79.10 ± 0.08^b^	2.12 ± 0.02^b^
40	77.41 ± 0.07^c^	2.01 ± 0.01^c^
60	72.59 ± 0.11^f^	1.35 ± 0.01^f^
80	74.95 ± 0.08^d^	1.53 ± 0.01^e^
100	73.73 ± 0.08^e^	1.69 ± 0.01^d^

Different letters in the data in the same column indicate significant differences (*P* < 0.05).

### Effect of high-pressure homogenization on functional properties of enzymatic hydrolyzed soybean protein concentrate

#### Analysis of solubility

**FIGURE 6:**
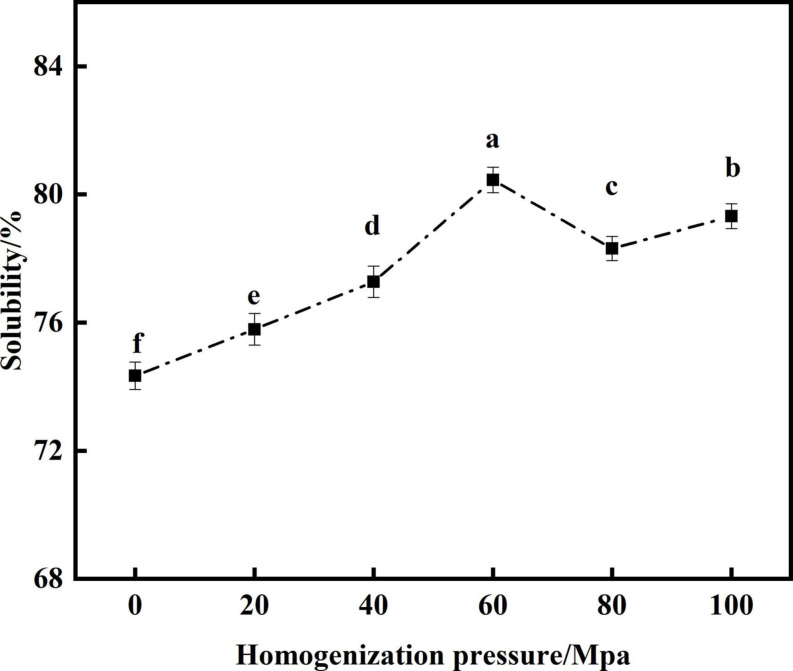
Effects of high-pressure homogenization (HPH) on the solubility of enzymatic hydrolyzed soybean protein concentrate (SPC).

Protein solubility is an important functional indicator for evaluating SPC (44). From Figure 6 that compared with the untreated samples after enzymatic hydrolysis, the HPH treatment significantly improved the solubility of SPC. With the increase of HP, the solubility showed a trend of first increase and decrease. When the HP was 60 Mpa, the solubility of SPC was the highest at 80.54%. This may be due to the HPH treatment, the secondary structure of protein is destroyed, the β-sheets content was decreased, and the random curl content was increased, so that the hydrophilic groups inside the protein are exposed to bind with water molecules, and the solubility of protein is increased (45, 46). When the HP is too high, the SPC molecules cross-link, the particle size increases, the specific surface area decreases, so that the binding sites between protein and water molecules become less, which leads to the decrease of solubility (47, 48). The results indicate that the HPH changes the molecular structure of SPC after enzymatic hydrolysis, the exposure degree of hydrophilic groups, the solution particle size and the specific surface area changed, and then the binding ability of water molecules changed, affecting the solubility of protein.

#### Analysis of water and oil holding capacity

**FIGURE 7:**
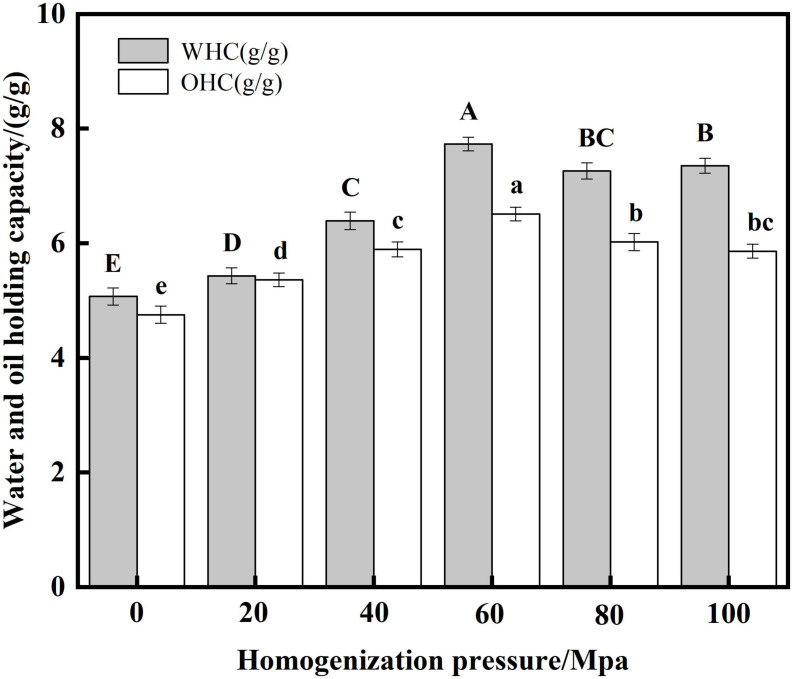
Effects of high-pressure homogenization (HPH) on the water and oil capacity of enzymatic hydrolyzed soybean protein concentrate (SPC).

Water-holding capacity (WHC) indicates interactions between proteins and water molecules in food systems (49). The level of oil holding capacity (OHC) of protein plays an important role in mixed or viscous foods (50). From Figure 7, the WHC and OHC of SPC were significantly increased by HPH compared with the untreated samples after enzymatic hydrolysis. With the increase of HP, the WHC and OHC of SPC increased first and then decreased. When the HP was 60 Mpa, the WHC and OHC of protein were 7.73 g/g and 6.51 g/g, respectively. This may be due to the HPH treatment destroys the protein structure and increases the exposure of charged amino acids and polar amino acids. Studies have shown that charged amino acid side chains have a strong potential to bind water molecules (51), thereby increasing the WHC. When the HP is too high, the WHC decreases, which may be due to the increased surface hydrophobicity of the exposed hydrophobic group, and the relatively high H_0_ limits the protein-water interaction (52). The increase in OHC may be due to the partial unfolding and denaturation of the protein’s surface exposing a large number of hydrophobic groups to help form an appropriate network to capture oil droplets (53). However, when the HP is too high, the protein β-sheets increases, which reduces the binding of protein and oil droplets, thus leading to the decrease of OHC. These results indicated that HPH could destroy the structure of enzymatic SPC, change the degree of group exposure, and change the WHC and OHC of protein.

#### Analysis of emulsifying activity and emulsifying stability

**FIGURE 8:**
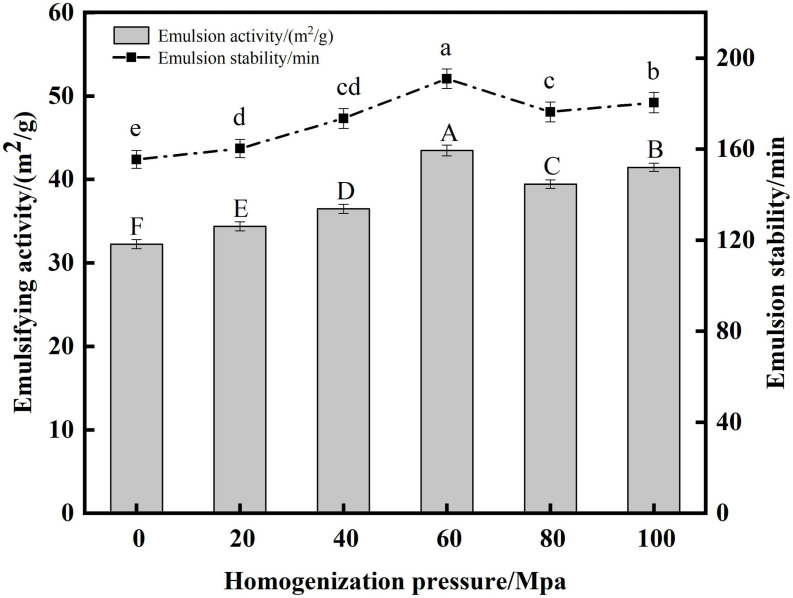
Effects of high-pressure homogenization (HPH) on the emulsifying activity (EA) and emulsifying stability (ES) of enzymatic hydrolyzed soybean protein concentrate (SPC).

Protein has emulsifying activity due to its hydrophilic and hydrophobic groups, and is an important emulsifier in food processing (54). EA indicates the ability of the protein to adsorb rapidly at the oil-water interface, and ES reflects the stability of the protein to stay at the water-oil interface over a period of time (27, 55). From Figure 8 the HPH treatment resulted in a significant increase in the EA and ES of the proteins compared to the untreated samples after enzymatic digestion. As the HP increased, the SPC emulsification activity and emulsion stability both tended to increase first and then decrease. When the HP was 60 Mpa, the EA and ES of protein were the highest at 43.46 m^2^/g and 190.35 min, respectively. This may be due to the fact that as the HP increases, the protein structure opens and more easily unfolds on the oil droplet surface to increase its flexibility, therefore the protein is more easily adsorbed on the oil droplet surface, thus increasing the ES (56). When the HP is too high, the absolute value of the protein potential decreases, the electrostatic repulsion between the proteins decreases, and protein aggregation is easily formed, which reduces the adsorption force on the oil-water interface, thereby reducing the stability of the emulsion and leading to the EA reduce (57). The results showed that the structure of SPC was changed by HPH, and its adsorption capacity to the oil-water interface was changed, thus its EA and ES were changed.

#### Analysis of confocal laser scanning microscope

**FIGURE 9:**
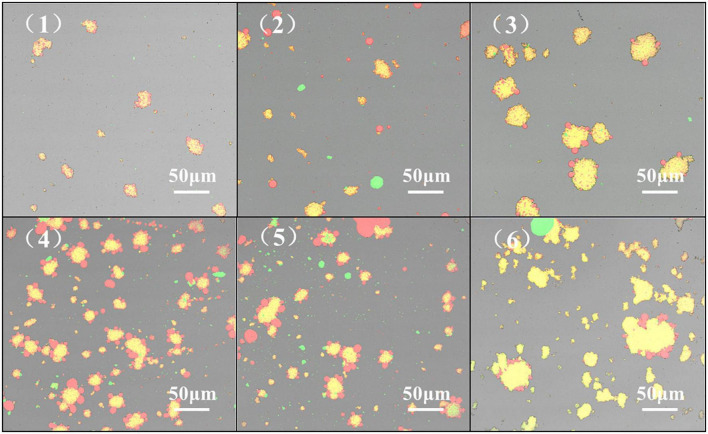
Effect of high-pressure homogenization (HPH) on confocal laser microscope scanning images of enzymatic hydrolyzed soybean protein concentrate (SPC). Note: (1-6) in the figure represent HPH treatment of 0, 20, 40, 60, 80, and 100 Mpa after enzymatic hydrolysis.

Confocal Laser Scanning Microscope to display the microscopic morphology and distribution of the emulsion. The emulsion droplets are evenly distributed, indicating that the emulsion system is stable, and the emulsion distribution is uneven and aggregated, indicating that the emulsion stability is deteriorated (58). From Figure 9, the HPH treatment resulted in a more uniform distribution of the emulsion droplets of proteins compared to the untreated samples after enzymatic digestion. With the increase of HP, the droplet distribution of SPC emulsion showed a state of first dispersion and then aggregation. When the HP is 60Mpa, the droplet distribution of SPC emulsion is the most uniform. This may be due to the fact that the HPH treatment destroys the structure of the protein, the particle size is reduced and the structure is opened and the hydrophobic groups are exposed. The droplet distribution is more stable (59). When the HP is too high, the protein emulsion will appear in the state of aggregation and flocculation. This may be because with the increase of HP, it may also cause reaggregation between emulsion droplets, resulting in the increase of emulsion droplet size and protein aggregation (60). The results indicated that the HPH changed the structure of SPC and the exposure degree of its intramolecular groups, thus changing the distribution of its emulsion droplets.

## Conclusion

In this paper, Alcalase protease and high-pressure homogenization (HPH) were used to modify soybean protein concentrate (SPC) to explore the effects on the physicochemical and functional properties of SPC. The results showed that the degree of hydrolysis of SPC was 4.1% and the antigen protein was degraded after Alcalase hydrolysis. From the physicochemical results of SPC, when the homogenization pressure (HP) was 60 MPa, the enzymatic SPC had the smallest particle size and the highest absolute zate potential value of 33.45 mV, the content of secondary structure β-sheet is the lowest, the content of random curl is the highest, and the surface The hydrophobicity (H_0_) is the highest, the size of protein fragments on the microstructure surface is the smallest and the largest, and the denaturation temperature (T_*d*_) and denaturation enthalpy (ΔH) are the lowest at 72.59°C and 1.35 J/g, respectively. This indicates that the enzymatic hydrolysis of SPC by HPH reduces the particle size of the solution, increases the absolute value of the potential, and the secondary structure becomes disordered and unstable, resulting in a decrease in T_*d*_ and ΔH. From the functional results of SPC, when the HP is 60 Mpa, the solubility of enzymatically hydrolyzed SPC is up to 80.54%, and the water and oil holding capacities are up to 7.73 g/g and 6.51 g/g, respectively. The emulsion activity and stability were the best at 43.46 m^2^/g and 190.35 min, and the emulsion droplet distribution was the most uniform. This indicates that the HPH treatment destroys the structure of enzymatic hydrolyzed SPC, changes its physicochemical properties, and improves its functional properties. In this study, SPC was modified by Alcalase protease and HPH combined treatment, which provided a theoretical basis for improving the functionality and application range of SPC, so that it could be used in high-value in the food field.

## Data availability statement

The original contributions presented in this study are included in the article/supplementary material, further inquiries can be directed to the corresponding author/s.

## Author contributions

YL: conceptualization and writing—original draft preparation. YL and YG: data curation. YZ: methodology and investigation. TC: supervision. YG and SL: writing—review and editing. LZ and ZG: funding acquisition. All authors have read and agreed to the published version of the manuscript.
